# Immunohistochemical Evaluation of Cathepsin B, L, and S Expression in Breast Cancer Patients

**DOI:** 10.1007/s11307-024-01955-5

**Published:** 2024-09-27

**Authors:** Daan G. J. Linders, Okker D. Bijlstra, Laura C. Fallert, N. Geeske Dekker-Ensink, Taryn L. March, Martin Pool, Ethan Walker, Brian Straight, James P. Basilion, Matthew Bogyo, Jacobus Burggraaf, Denise E. Hilling, Alexander L. Vahrmeijer, Peter J. K. Kuppen, A. Stijn L. P. Crobach

**Affiliations:** 1 Department of Surgery, Leiden University Medical Center, 2333 ZA Leiden, The Netherlands; 2 Department of Clinical Pharmacy and Toxicology, Leiden University Medical Center, 2333 ZA Leiden, The Netherlands; 3 Department of Biomedical Engineering, Case Western Reserve University, Cleveland, OH 44106 USA; 4 Akrotome Imaging Inc, Charlotte, NC 28205 USA; 5 Department of Radiology, Case School of Medicine, Case Western Reserve University, Cleveland, OH 44106 USA; 6 Department of Pathology, Stanford University School of Medicine, Stanford, CA USA; 7 Department of Microbiology and Immunology, Stanford University School of Medicine, Stanford, CA USA; 8 Centre for Human Drug Research, 2333 AL Leiden, The Netherlands; 9 Leiden Academic Center for Drug Research, Leiden University, 2300 RA Leiden, The Netherlands; 10 Department of Surgical Oncology and Gastrointestinal Surgery, Erasmus MC Cancer Institute, University Medical Center Rotterdam, 3015 GD Rotterdam, The Netherlands; 11 Department of Pathology, Leiden University Medical Center, 2333 ZA Leiden, The Netherlands

**Keywords:** Cysteine cathepsins, Breast cancer, Target expression, Optical surgical navigation, Fluorescence-guided surgery, Activatable fluorescent imaging agents

## Abstract

**Purpose:**

Cysteine cathepsins are proteases that play a role in normal cellular physiology and neoplastic transformation. Elevated expression and enzymatic activity of cathepsins in breast cancer (BCa) indicates their potential as a target for tumor imaging. In particular cathepsin B (CTSB), L (CTSL), and S (CTSS) are used as targets for near-infrared (NIR) fluorescence imaging (FI), a technique that allows real-time intraoperative tumor visualization and resection margin assessment. Therefore, this immunohistochemical study explores CTSB, CTSL, and CTSS expression levels in a large breast cancer patient cohort, to investigate in which BCa patients the use of cathepsin-targeted NIR FI may have added value.

**Procedures:**

Protein expression was analyzed in tumor tissue microarrays (TMA) of BCa patients using immunohistochemistry and quantified as a total immunostaining score (TIS), ranging from 0–12. In total, the tissues of 557 BCa patients were included in the TMA.

**Results:**

CTSB, CTSL, and CTSS were successfully scored in respectively 340, 373 and 252 tumors. All tumors showed CTSB, CTSL, and/or CTSS expression to some extent (TIS > 0). CTSB, CTSL, and CTSS expression was scored as high (TIS > 6) in respectively 28%, 80%, and 18% of tumors. In 89% of the tumors scored for all three cathepsins, the expression level of one or more of these proteases was scored as high (TIS > 6). Tumors showed significantly higher cathepsin expression levels with advancing Bloom-Richardson grade (*p* < 0.05). Cathepsin expression was highest in estrogen receptor (ER)-negative, human epidermal growth factor receptor 2(HER2)-positive and triple-negative (TN) tumors. There was no significant difference in cathepsin expression between tumors that were treated with neoadjuvant systemic therapy and tumors that were not.

**Conclusions:**

The expression of at least one of the cysteine cathepsins B, L and S in all breast tumor tissues tested suggests that cathepsin-activatable imaging agents with broad reactivity for these three proteases will likely be effective in the vast majority of breast cancer patients, regardless of molecular subtype and treatment status. Patients with high grade ER-negative, HER2-positive, or TN tumors might show higher imaging signals.

## Introduction

Breast cancer is the most frequently diagnosed cancer and the leading cause of cancer-related mortality in women worldwide [1]. Most breast cancer patients are treated with breast-conserving surgery (BCS) combined with adjuvant radiotherapy [2]. To prevent local tumor recurrence and associated additional treatments, it is of paramount importance to achieve tumor-negative resection margins during BCS [3]. However, BCS currently still results in a tumor-positive resection margin in 10–40% of patients [4, 5]. Those patients need to undergo re-resection or additional boost radiotherapy, resulting in worse cosmetic outcomes and increased complication risks, morbidity and healthcare costs [2, 6, 7]. Numerous imaging and pathology methods have been investigated to reduce tumor-positive resection margin rates, yet most have significant technical or clinical limitations that have precluded widespread adoption [8].

A technique that shows promise is intraoperative tumor-targeted near-infrared (NIR) fluorescence imaging (FI). It combines the administration of an imaging contrast agent, comprising a fluorophore and a targeting moiety, with the use of a fluorescence-sensitive camera, matched to operate in the range of NIR fluorescence light (700–900 nm) [9]. It allows real-time optical imaging to selectively highlight cells that (over)express certain molecular targets. It can be used to detect residual tumor in the surgical cavity and guide additional resection to achieve a tumor-free margin during BCS.

To date, various imaging agents for fluorescence-guided BCS targeting different molecules have been investigated pre-clinically and in breast cancer patients [10]. However, only cysteine cathepsin-targeted imaging agents, the most developed and extensively investigated subgroup of imaging agents in breast cancer, have been shown to enable visualization of (residual) tumor tissue in the surgical cavity during BCS [11]. Recently, the U.S. Food and Drug Administration (FDA) has approved the cathepsin-activatable imaging agent pegulicianine (also known as Lumisight or LUM015) in adults with breast cancer to assist in the detection of cancerous tissue during BCS [12, 13].

Cysteine cathepsins are an important group of proteolytic enzymes that play a major role in neoplastic transformation [14]. Various members of the cysteine cathepsin family are highly overexpressed and have an increased enzymatic activity in breast cancer tissues compared with healthy breast tissue, making these enzymes suitable targets for fluorescence-guided BCS [11, 15–17, 18]. Notably, most existing cathepsin-targeted fluorescent imaging agents are directed against cathepsin B, L, and/or S [11]. This is also true for pegulicianine, which is activated by cathepsin B, L, K, and S [19]. These imaging agents often are fluorescently quenched and become activated upon covalent binding to or enzymatic cleavage by cathepsins. Although it has been shown that cathepsin B, L and S have elevated expression levels and enzymatic activity in breast cancer and that this upregulation correlates with an increased imaging agent activation, the association between their upregulation and relevant clinicopathological characteristics of breast cancer patients, such as histology, grade, molecular subtype and neoadjuvant treatment status, is not well defined and has only been investigated in relatively small patient cohorts[15–17, 20–23]. Therefore, this immunohistochemical study explores cathepsin B, L, and S expression levels in a large breast cancer patient cohort, to investigate in which breast cancer patients the use of cathepsin-targeted NIR FI may have added value.

## Patients and Methods

### Human Breast Cancer Tissue Samples

The study population consisted of the PRIMA cohort; female patients diagnosed with breast cancer who underwent surgery at the Leiden University Medical Center (LUMC) between 1997 and 2009 [24]. For these patients, pre-cut tissue microarray (TMA) slides containing tumor tissue punches were available and collected. Available data for these patients included age at diagnosis, tumor morphology, grade, dimensions, receptor status and local and systemic therapy. Not all clinical and pathology data was available for each patient.

### Immunohistochemistry (IHC)

TMA slides were deparaffinized using xylene and rehydrated in decreasing concentrations of ethanol. Subsequently, slides were rinsed with demineralized water, and endogenous peroxidase was blocked with 0.3% hydrogen peroxidase (Merck Millipore, Darmstadt, Germany) for 20 min at room temperature. Slides were rinsed with demineralized water, and antigen retrieval was performed in the PT Link (Agilent, Santa Clara, CA, USA), using either Target Retrieval Solution Low pH (cathepsin B and L) or High pH (cathepsin S) at 95 ◦C for 10 min. After rinsing with phosphate-buffered saline (PBS, pH 7.4), slides were incubated overnight at room temperature with primary antibodies, diluted in 1% bovine serum albumin/PBS, against cathepsin B (clone ab125067, Abcam, 0.263 mg/mL, dilution 1:50), cathepsin L (clone ab6314, Abcam, 1 mg/mL, dilution 1:1600), and cathepsin S (clone ab134157, Abcam, 0.226 mg/mL, dilution 1:500). After three PBS washing steps, the slides were incubated with a horseradish peroxidase (HRP)-labelled secondary antibody against mouse or rabbit (EnVision, Agilent, Santa Clara, USA) for 30 min at room temperature. Binding of the antibody was visualized using liquid DAB + substrate chromogen system (Agilent, Santa Clara, USA). All slides were counterstained with hematoxylin for 10–15 s, dehydrated at 37 ◦C, and mounted with Pertex. Slides were digitized by scanning with the Philips IntelliSite Pathology Solution (Philips Electronics, Eindhoven, The Netherlands).

### Scoring Method

All TMA slides were scored for cathepsin B, L, and S expression by a board-certified pathologist (S.C.). Not all tumors were scored for all three cathepsins due to incidental poor tissue slice quality. The total immunostaining score (TIS) of each cathepsin was calculated for each tumor by multiplying the proportion score (PS) by the intensity score (IS) [15]. The PS represented the percentage of positively stained cells in the punch and ranged between 0 and 4 (0 = none; 1 0–10%; 2 = 11–50%; 3 = 51–80%; 4 > 80%). The IS represented the intensity of the stained cells in the punch and could range between 0 and 3 (0 = no staining; 1 = weak; 2 = moderate; 3 = strong). Subgroups were defined based on the calculated TIS: 0, no expression; 1–5, weak expression; 6–8, moderate expression; 9–12, intense expression. For dichotomization of the subgroups, TIS 0–5 (indicating no to weak expression) was categorized as low expression, and TIS 6–12 (indicating moderate-to-intense expression), categorized as high expression. The pathologist was blinded for the clinicopathological data of the tissues.

### Statistics

Statistical analyses were performed using SPSS version 25.0 software (SPSS, IBM Corporation, NY, USA) and GraphPad Prism 9 (GraphPad Software Inc., La Jolla, CA, USA).

Descriptive statistics are depicted as mean (SD) or median (IQR). Differences in TIS of an individual cathepsin type between tumors with different morphology or receptor status were investigated using the Mann–Whitney test. The Kruskal–Wallis test was used to investigate differences in clinicopathological characteristics between the groups of patients of whom the tissue was scored for the expression of a specific cathepsin. The same test was performed to define differences in TIS of an individual cathepsin between tumors of different grade. To investigate the association between the expression of two cathepsins in the same tumor, the Spearman’s correlation coefficient was calculated. All statistical tests were performed two sided and results were considered statistically significant at the level of p < 0.05. Data are summarized in graphs and bar charts generated by GraphPad Prism 9.

## Results

### Patient and Tumor Characteristics

In total, the tumor punches of 557 breast cancer patients were included. Cathepsin B, L, and S expression could be successfully scored in respectively 340, 373 and 252 of these tumors—due to incidental poor tissue section quality. The clinicopathological characteristics of all patients are summarized in Table 1 (percentages are corrected for *unknowns*)*.* Median age at diagnosis was 55 (IQR 47–66). Most patients (79.9%) had an invasive carcinoma no special type (NST). Tumors showed estrogen receptor (ER), progesterone receptor (PR) and human epidermal growth factor receptor 2 (HER2) overexpression in respectively 75%, 55%, and 24% of patients. Tumors were Bloom-Richardson grade 1, 2, and 3 in respectively 17%, 45%, and 38% of cases. Median tumor size was 19 mm (IQR 13–27). The majority of patients (92.6%) did not receive neoadjuvant treatment. There were no significant differences in clinicopathological characteristics between the subgroups of patients of whom the tissue was scored for a certain cathepsin (*p* > 0.05).

**Table 1:** Patient and tumor characteristics

	All patients (*n* = *557)*	Cathepsin B (*n* = *340)*	Cathepsin L (*n* = *373)*	Cathepsin S (*n* = *252)*	*P* Value
Age at diagnosis, median years(range)	55 (21–89)	55 (21–89)	53 (21–89)	57 (24–89)	0.150
Tumor morphology					
*Invasive carcinoma NST*	445 (79.9%)	269 (79.1%)	307 (82.2%)	195 (77.4%)	
*Invasive lobular carcinoma*	54 (9.7%)	35 (10.3%)	28 (7.5%)	32 (12.7%)	
*Invasive ductal and lobular carcinoma*	19 (3.4%)	12 (3.5%)	11 (2.9%)	10 (4%)	0.094
*Mucinous carcinoma*	5 (0.9%)	4 (1.2%)	3 (0.8%)	2 (0.8%)	
*Adenocarcinoma NOS*	11 (2%)	11 (3.2%)	8 (2.2%)	4 (1.6%)	
*Other*	23 (4.1%)	9 (2.7%)	16 (4.4%)	9 (3.5%)	
Receptor status					
*ER* + */ unknown*	371 (74.9%) / 62 (11.1%)	225 (78.4%) / 53 (15.6%)	240 (71.9%) / 39 (10.5%)	190 (77.6%) / 7 (2.8%)	0.112
*PR* + */ unknown*	261 (54.6%) / 79(14.2%)	152 (55.9%) / 68(20%)	179 (54.7%) / 46(12.3%)	116 (48.5%) / 13(5.2%)	0.206
*HER2* + */ unknown*	68 (24.3%) / 278(49.9%)	24 (25%) / 244(71.8%)	58 (24.7%) / 138(37%)	46 (27.9%) / 87(34.5%)	0.766
Tumor grade					
*1*	83 (16.9%)	52 (18%)	54 (16.1%)	38 (16.8%)	
*2*	220 (44.8%)	141 (48.8%)	140 (41.8%)	114 (50.5%)	
*3*	188 (38.3%)	96 (33.2%)	141 (42.1%)	74 (32.7%)	0.088
*Unknown*	66 (11.8%)	51 (15%)	38 (10.2%)	26 (10.3%)	
Neoadjuvant therapy					
*No*	516 (92.8%)	320 (94.4%)	336 (90.3%)	228 (90.5%)	
*Chemotherapy*	20 (3.6%)	9 (2.7%)	16 (4.3%)	8 (3.2%)	
*Hormonal therapy*	19 (3.4%)	9 (2.7%)	19 (5.1%)	15 (6%)	0.120
*Both*	1 (0.2%)	1 (0.3%)	1 (0.3%)	1 (0.4%)	
*Unknown*	1 (0.2%)	1 (0.3%)	1 (0.3%)	0 (0%)	
Tumor size, median mm (range)	19 (1–150)	20 (1–150)	19 (3–120)	19 (3–150)	0.684
Tumor-positive lymph nodes					
*Yes*	226 (42.8%)	132 (41.8%)	161 (45.1%)	106 (42.9%)	0.652
*Unknown*	29 (5.2%)	24 (7.1%)	16 (4.3%)	5 (2%)	

Shown are the patient and tumor characteristics of all tumors included in the study (*n* = 557) and of the subgroups of tumors scored for cathepsin B (*n* = 340), cathepsin L (*n* = 373), and cathepsin S (*n* = 252). The reported percentages are corrected for *unknowns*. No significant differences in characteristics were found between the subgroups of patients of whom the tissue was scored for a certain cathepsin (*p* > 0.05). **Abbreviations:** ER, estrogen receptor; HER2, human epidermal growth factor receptor 2; NOS, not otherwise specified; NST, no special type; PR, progesterone receptor

### High Versus Low Expression of Cathepsin B, L, And S in Breast Cancer Tissue

**Fig. 1:**
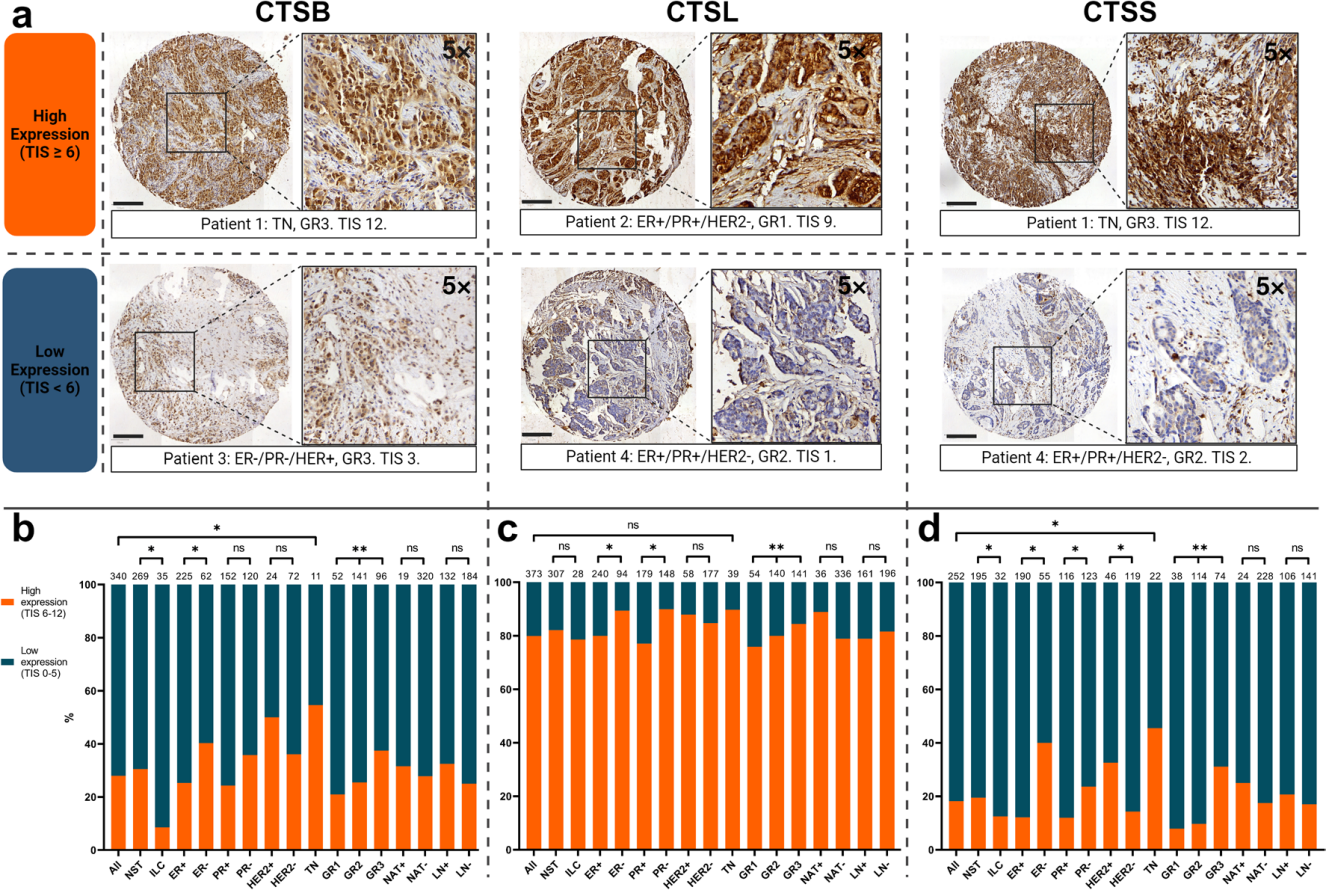
**(a) **Representative images of cathepsin B, L, and S stained TMA punches of breast tumors. Two tumors are shown for each cathepsin: in the upper panel one with a high expression (TIS ≥ 6) and in the lower panel one with a low expression (TIS < 6). The scale bar represents 200 µm in the whole punch. The zoom area represents a 5 × magnification of the corresponding area in the whole punch. **(b-d)** High and low expression of respectively cathepsin B (**b)**, L **(c)**, and S **(d)** in breast tumors. In total, 340, 373, and 252 tumors were successfully analyzed for cathepsin B, L, and S expression, respectively. Shown are the percentages of tumors with certain clinicopathological characteristics that had a high (TIS ≥ 6) and low (TIS < 6) cathepsin expression. The number of tumors in each category is shown above each bar. Cathepsin B expression was significantly higher in invasive carcinomas NST compared with invasive lobular carcinomas, in ER-negative compared with ER-positive tumors, in triple-negative tumors compared with the other molecular subtypes and in higher grade tumors compared with lower grade tumors (*p* < 0.05). Cathepsin L expression was significantly higher in ER-negative tumors compared with ER-positive tumors, in PR-negative tumors compared with PR-positive tumors and in higher grade tumors compared with lower grade tumors (*p *< 0.05). Cathepsin S expression was significantly higher in invasive carcinomas NST compared with invasive lobular carcinomas, in ER-negative compared with ER-positive tumors, in PR-negative compared with PR-positive tumors, in HER2-positive compared with HER2-negative tumors, in triple-negative tumors compared with the other molecular subtypes and in higher grade tumors compared with lower grade tumors (*p* < 0.05). **Abbreviations:** CTSB, cathepsin B; CTSL, cathepsin L; CTSS, cathepsin S; ER, estrogen receptor; GR, grade; HER2, human epidermal growth factor receptor 2; ILC, invasive lobular carcinoma; LN, lymph-node; NAT, neoadjuvant therapy; NST, no special type; PR, progesterone receptor; TIS, total immunostaining score; TN, triple-negative. * = Mann–Whitney test *p* < 0.05. ** = Kruskal–Wallis test *p* < 0.05.

Figure 1a shows representative TMA punches immunohistochemically stained for cathepsin B, L, and S of tumors with respectively a high and low expression of these proteases, derived from four separate patients. All three cathepsins were expressed by breast cancer cells and cells in the stroma. Cathepsin B and L mainly showed expression by breast cancer cells. Cathepsin S was mainly expressed by cells in the stroma.

#### Cathepsin B Expression Levels

TIS of cathepsin B are summarized in Table 2. Of the total of 340 breast tumors that were successfully analyzed for cathepsin B, 95 (28%) showed a high (TIS 6–12) and 245 (72%) a low (TIS 0–5) expression. Only two tumors showed no cathepsin B expression. Cathepsin B expression was not limited to specific morphologic or molecular subtypes.

**Table 2:** Cathepsin B total immunostaining scores (TIS) of immunohistochemically stained breast cancer punches

CTSB	No expression (TIS 0)	Weak expression (TIS 1–5)	Moderate expression (TIS 6–8)	Intense expression (TIS 9–12)	Total punches
All patients	2 (0.6%)	243 (71.4%)	80 (23.6%)	15 (4.4%)	340
Invasive carcinoma NST	1 (0.4%)	186 (69.1%)	71 (26.4%)	11 (4.1%)	269
Invasive lobular carcinoma	0 (0%)	32 (91.4%)	2 (5.7%)	1 (2.9%)	35
Invasive ductal and lobular carcinoma	0 (0%)	11 (91.7%)	1 (8.3%)	0 (0%)	12
Mucinous carcinoma	1 (25%)	2 (50%)	0 (0%)	1 (25%)	4
Adenocarcinoma NOS	0 (0%)	6 (54.5%)	3 (27.3%)	2 (18.2%)	11
Other	0 (0%)	6 (66.7%)	3 (33.3%)	0 (0%)	9
ER +	0 (0%)	168 (74.7%)	49 (21.7%)	4 (3.6%)	225
ER-	0 (0%)	37 (59.7%)	19 (30.6%)	6 (9.7%)	62
PR +	0 (0%)	115 (75.7%)	31 (20.3%)	6 (4%)	152
PR-	0 (0%)	77 (64.2%)	35 (29.2%)	8 (6.6%)	120
HER2 +	0 (0%)	12 (50%)	9 (37.5%)	3 (12.5%)	24
HER2-	0 (0%)	46 (63.9%)	23 (31.9%)	3 (4.2%)	72
Triple Negative (ER-/PR-/HER2-)	0 (0%)	5 (45.4%)	5 (45.5%)	1 (9.1%)	11
Grade 1	0 (0%)	41 (79%)	9 (17.3%)	2 (3.7%)	52
Grade 2	1 (0.7%)	104 (73.8%)	30 (21.2%)	6 (4.3%)	141
Grade 3	0 (0%)	60 (62.5%)	30 (31.3%)	6 (6.2%)	96
Tumor > 1 mm but ≤ 20 mm	2 (1.1%)	128 (70.7%)	45 (24.9%)	6 (3.3%)	181
Tumor > 20 mm but ≤ 50 mm	0 (0%)	101 (73.2%)	28 (20.3%)	9 (6.5%)	138
Tumor > 50 mm	0 (0%)	10 (62.5%)	6 (37.5%)	0 (0%)	16
NAT	0 (0%)	13 (68.4%)	5 (26.3%)	1 (5.3%)	19
No NAT	2 (0.6%)	229 (71.6%)	75 (23.4%)	14 (4.4%)	320
Tumor-positive lymph nodes	0 (0%)	89 (67.5%)	37 (28%)	6 (4.5%)	132
No tumor-positive lymph nodes	2 (1.1%)	136 (73.9%)	39 (21.2%)	7 (3.8%)	184

Displayed are the total immunostaining scores (TIS) of all breast cancer punches immunohistochemically stained and successfully scored for cathepsin B, for different patient and tumor characteristics

**Abbreviations:** CTSB, cathepsin B; ER, estrogen receptor; HER2, human epidermal growth factor receptor 2; NAT, neoadjuvant therapy; NOS, not otherwise specified; NST, no special type; PR, progesterone receptor; TIS, total immunostaining score

Figure 1b shows the percentages of breast tumors with a high and low expression of cathepsin B, for different patient and tumor characteristics. Cathepsin B expression was significantly higher in invasive carcinomas NST compared with invasive lobular carcinomas (high in 30.5% vs. 8.6%, *p* < 0.05). ER-negative tumors had a significantly higher cathepsin B expression compared with ER-positive tumors (high in 40.3% vs. 25.3%, *p* < 0.05). Compared to the other molecular subtypes, triple-negative (TN) tumors had a significantly higher cathepsin B expression (high in 54.6% vs. 27.1%, *p* < 0.05). Additionally, cathepsin B expression significantly increased with advancing grade (high in 21% vs. 25.5% vs. 37.5%, *p* < 0.05).

There was no significant difference in cathepsin B expression between PR-positive and -negative tumors (*p* = 0.103) or between HER2-positive and- negative tumors (*p *= 0.131). Furthermore, cathepsin B expression did not differ significantly between the tumors of patients who received neoadjuvant therapy and patients who did not (*p* = 0.773) or between patients who had tumor-positive lymph nodes and patients who did not (*p* = 0.213).

### Cathepsin L Expression Levels

TIS of cathepsin L are summarized in Table 3. Of the total of 373 breast tumors that were successfully analyzed for cathepsin L, 298 (80%) showed a high (TIS 6–12) and 75 (20%) a low (TIS 0–5) expression. There were no tumors without any cathepsin L expression.

**Table 3:** Cathepsin L total immunostaining scores (TIS) of immunohistochemically stained breast cancer punches

CTSL	No expression (TIS 0)	Weak expression (TIS 1–5)	Moderate expression (TIS 6–8)	Intense expression (TIS 9–12)	Total punches
All patients	0 (0%)	75 (20.1%)	69 (18.5%)	229 (61.4%)	373
Invasive carcinoma NST	0 (0%)	55 (17.9%)	58 (18.9%)	194 (63.2%)	307
Invasive lobular carcinoma	0 (0%)	6 (21.4%)	5 (17.9%)	17 (60.7%)	28
Invasive ductal and lobular carcinoma	0 (0%)	3 (27.3%)	1 (9.1%)	7 (63.6%)	11
Mucinous carcinoma	0 (0%)	2 (66.7%)	1 (33.3%)	0 (0%)	3
Adenocarcinoma NOS	0 (0%)	4 (50%)	0 (0%)	4 (50%)	8
Other	0 (0%)	5 (31.3%)	4 (25%)	7 (43.7%)	16
ER +	0 (0%)	48 (20%)	44 (18.3%)	148 (61.7%)	240
ER-	0 (0%)	10 (10.6%)	18 (19.2%)	66 (70.2%)	94
PR +	0 (0%)	41 (22.9%)	35 (19.6%)	103 (57.5%)	179
PR-	0 (0%)	15 (10.1%)	25 (16.9%)	108 (73%)	148
HER2 +	0 (0%)	7 (12.1%)	12 (20.7%)	39 (67.2%)	58
HER2-	0 (0%)	27 (15.3%)	27 (15.3%)	123 (69.4%)	177
Triple Negative (ER-/PR-/HER2-)	0 (0%)	4 (10.3%)	9 (23.1%)	26 (66.6%)	39
Grade 1	0 (0%)	13 (24.1%)	18 (33.3%)	23 (42.6%)	54
Grade 2	0 (0%)	28 (20%)	23 (16.4%)	89 (63.6%)	140
Grade 3	0 (0%)	22 (15.6%)	22 (15.6%)	97 (68.8%)	141
Tumor > 1 mm but ≤ 20 mm	0 (0%)	47 (23.4%)	34 (16.9%)	120 (59.7%)	201
Tumor > 20 mm but ≤ 50 mm	0 (0%)	24 (16%)	29 (19.3%)	97 (64.7%)	150
Tumor > 50 mm	0 (0%)	3 (15.8%)	4 (21.1%)	12 (63.1%)	19
NAT	0 (0%)	4 (11.1%)	3 (8.3%)	29 (80.6%)	36
No NAT	0 (0%)	71 (21.1%)	65 (19.4%)	200 (59.5%)	336
Tumor-positive lymph nodes	0 (0%)	34 (21.1%)	24 (14.9%)	103 (64%)	161
No tumor-positive lymph nodes	0 (0%)	36 (18.4%)	42 (21.4%)	118 (60.2%)	196

Displayed are the total immunostaining scores (TIS) of all breast cancer punches immunohistochemically stained and successfully scored for cathepsin L, for different patient and tumor characteristics

**Abbreviations:** CTSL, cathepsin L; ER, estrogen receptor; HER2, human epidermal growth factor receptor 2; NAT, neoadjuvant therapy; NOS, not otherwise specified; NST, no special type; PR, progesterone receptor; TIS, total immunostaining score

Figure 1c shows the percentages of breast tumors with a high and low expression of cathepsin L, for different patient and tumor characteristics. Cathepsin L expression was significantly higher in ER-negative tumors compared with ER-positive tumors (high in 89.4% vs. 80%, *p* < 0.05) and in PR-negative tumors compared with PR-positive tumors (high in 89.9% vs. 77.1%, *p* < 0.05). Additionally, cathepsin L significantly increased with advancing grade (high in 75.9% vs. 80% vs. 84.4%, *p* < 0.05).

There was no significant difference in cathepsin L expression between invasive carcinomas NST and invasive lobular carcinomas (*p* = 0.281) or between TN tumors and the other molecular subtypes (*p* = 0.177). Expression of cathepsin L did not differ significantly between HER2-positive and -negative tumors (*p* = 0.682). Furthermore, there was no significant difference in cathepsin L expression between the tumors of patients who received neoadjuvant therapy and patients who did not (*p* = 0.164) or between patients who had tumor-positive lymph nodes and patients who did not (*p* = 0.723).

### Cathepsin S Expression Levels

Total immunostaining scores (TIS) of cathepsin S are summarized in Table 4. Of the total of 252 breast tumors that were successfully analyzed for cathepsin S, 46 (18.2%) showed a high (TIS 6–12) and 206 (81.8%) a low (TIS 0–5) expression. Four tumors showed no expression. Cathepsin S expression was not limited to specific morphologic or molecular subtypes.

**Table 4:** Cathepsin S total immunostaining scores (TIS) of immunohistochemically stained breast cancer punches

CTSS	No expression (TIS 0)	Weak expression (TIS 1–5)	Moderate expression (TIS 6–8)	Intense expression (TIS 9–12)	Total punches
All patients	4 (1.6%)	202 (80.2%)	33 (13.1%)	13 (5.1%)	252
Invasive carcinoma NST	3 (1.5%)	154 (79%)	27 (13.8%)	11 (5.7%)	195
Invasive lobular carcinoma	0 (0%)	28 (87.5%)	4 (12.5%)	0 (0%)	32
Invasive ductal and lobular carcinoma	1 (10%)	9 (90%)	0 (0%)	0 (0%)	10
Mucinous carcinoma	0 (0%)	2 (100%)	0 (0%)	0 (0%)	2
Adenocarcinoma NOS	0 (0%)	3 (75%)	1 (25%)	0 (0%)	4
Other	0 (0%)	6 (66.7%)	1 (11.1%)	2 (22.2%)	9
ER +	4 (2.1%)	163 (85.8%)	19 (10%)	4 (2.1%)	190
ER-	0 (0%)	33 (60%)	13 (23.6%)	9 (16.4%)	55
PR +	4 (3.5%)	98 (84.5%)	12 (10.3%)	2 (1.7%)	116
PR-	0 (0%)	94 (76.4%)	18 (14.6%)	11 (9%)	123
HER2 +	1 (2.2%)	30 (65.2%)	10 (21.7%)	5 (10.9%)	46
HER2-	3 (2.5%)	99 (83.2%)	11 (9.2%)	6 (5.1%)	119
Triple Negative (ER-/PR-/HER2-)	0 (0%)	12 (54.5%)	4 (18.2%)	6 (27.3%)	22
Grade 1	0 (0%)	35 (92.1%)	2 (5.3%)	1 (2.6%)	38
Grade 2	3 (2.6%)	100 (87.7%)	10 (8.8%)	1 (0.9%)	114
Grade 3	0 (0%)	51 (68.9%)	15 (20.3%)	8 (10.8%)	74
Tumor > 1 mm but ≤ 20 mm	3 (2.1%)	112 (80%)	16 (11.4%)	9 (6.4%)	140
Tumor > 20 mm but ≤ 50 mm	1 (1.1%)	73 (77.7%)	16 (17%)	4 (4.2%)	94
Tumor > 50 mm	0 (0%)	16 (94.1%)	1 (5.9%)	0 (0%)	17
NAT	1 (4.2%)	17 (70.8%)	4 (16.7%)	2 (8.3%)	24
No NAT	3 (1.3%)	185 (81.2%)	29 (12.7%)	11 (4.8%)	228
Tumor-positive lymph nodes	2 (1.9%)	82 (77.4%)	14 (13.2%)	8 (7.5%)	106
No tumor-positive lymph nodes	2 (1.4%)	115 (81.6%)	19 (13.5%)	5 (3.5%)	141

Displayed are the total immunostaining scores (TIS) of all breast cancer punches immunohistochemically stained and successfully scored for cathepsin S, for different patient and tumor characteristics

**Abbreviations:** CTSS, cathepsin S; ER, estrogen receptor; HER2, human epidermal growth factor receptor 2; NAT, neoadjuvant therapy; NOS, not otherwise specified; NST, no special type; PR, progesterone receptor; TIS, total immunostaining score

Figure 1d shows the percentages of breast tumors with a high and low expression of cathepsin S, for different patient and tumor characteristics. Cathepsin S expression was significantly higher in invasive carcinomas NST compared with invasive lobular carcinomas (high in 19.5% vs. 12.5%, *p* < 0.05). ER-negative tumors had a significantly higher cathepsin S expression compared with ER-positive tumors (high in 40% vs. 12.1%, *p* < 0.05). Similarly, PR-negative tumors had a significantly higher cathepsin S expression compared with PR-positive tumors (high in 23.6% vs. 12%, *p* < 0.05). Contrarily, HER2-positive tumors had a significantly higher cathepsin S expression compared with HER2-negative tumors (high in 32.6% vs. 14.3%, *p* < 0.05). Compared to the other molecular subtypes, TN tumors had a significantly higher cathepsin S expression (high in 45.5% vs. 15.7%, *p* < 0.05). Additionally, cathepsin S expression significantly increased with advancing grade (high in 21% vs. 25.5% vs. 37.5%, *p* < 0.05).

There was no significant difference in cathepsin S expression between the tumors of patients who received neoadjuvant therapy and patients who did not (*p* = 0.467) or between patients who had tumor-positive lymph nodes and patients who did not (*p* = 0.929).

### Association of Cathepsin B, L, and S Expression Within the Same Breast Tumor

Due to incidental poor tissue section quality, not all tumors could be analyzed for the expression of each cathepsin. Of all 557 included tumors, 173 were successfully stained and analyzed for both cathepsin B and L, 149 were successfully stained and analyzed for both cathepsin B and S, 132 were successfully stained and analyzed for both cathepsin L and S, and 46 were successfully stained and analyzed for all three cathepsins.

All tumors showed cathepsin B, L, and/or S expression to some extent (TIS > 0). In 41 (89%) of the 46 tumors scored for all three cathepsins, the expression level of one or more of these proteases was scored as high (TIS > 6). A weak but significant correlation was found between the expression levels of cathepsin B and L (*ρ* = 0.18, *p *< 0.05), cathepsin B and S (*ρ* = 0.33, *p* < 0.01), and cathepsin L and S (*ρ *= 0.31, *p* < 0.01).

## Discussion

Cathepsin-targeted NIR FI during BCS can be used to detect residual tumor in the surgical cavity and guide additional resection, to minimize tumor-positive resection margin rates and thereby the need for additional therapy. Most of the existing cathepsin-activatable fluorescent imaging agents are directed against cathepsin B, L, and/or S. Therefore, this immunohistochemical study investigated the expression of these cathepsins in TMAs of a large cohort of breast cancer patients. Semi-quantitative analysis of the stained breast cancer tissues showed all tumors had cathepsin B, L, and/or S expression to some extent (TIS > 0) and expression levels of cathepsin B, L, and S were high (TIS > 6) in 28%, 80%, and 18% of cases, respectively. In 89% of the tumors scored for all three cathepsins, the expression level of one or more of these proteases was scored as high. All three cathepsins were expressed by breast cancer cells, though not to the same extent. Mainly cathepsin B and L showed expression by breast cancer cells. Consistent with the literature, we found cathepsin B and L were also expressed by cells in the stroma, although to a lesser extent [20]. Cathepsin S was mainly expressed by cells in the stroma. Tumors showed significantly higher cathepsin expression levels with advancing Bloom-Richardson grade, confirming previous studies [21, 23]. Cathepsin expression was highest in ER-negative, HER2-positive and TN tumors. This aligns with findings from preclinical studies demonstrating cathepsin expression levels correlate with tumor aggressiveness and, interestingly, probe activation and fluorescence signal intensities in vivo [25–27].

To date, approximately 20% of breast cancer patients receive neoadjuvant systemic therapy, as it allows testing the sensitivity of a patient’s tumor for these systemic treatments and often makes less extensive surgery possible [28, 29]. For successful use of cathepsin-targeted NIR FI in these patients, the expression of cathepsins in tumor tissue must be maintained after neoadjuvant systemic therapy. In the current study, there was no significant difference in the expression level of cathepsin B, L, and S between tumors treated with neoadjuvant systemic therapy and tumors that were not, suggesting this technique could also be applied in patients undergoing breast-conserving surgery after neoadjuvant systemic therapy.

A common challenge with tumor-specific fluorescence-guided surgery is the lack of expression of a certain target in the tumors of a significant percentage of patients – also known as intertumoral heterogeneity. This can result in the absence of a fluorescence signal in the residual tumor tissue, i.e., a false negative [30]. Other important targets under investigation for fluorescence-guided breast cancer surgery, such as vascular endothelial growth factor receptor-A (VEGF-A), folate receptor α (FRα), and integrins, are only (over)expressed in approximately 70%, 50%, and 20% of breast cancer patients, respectively [10, 31–33]. In contrast, all breast tumors in this study express cathepsin B, L, and/or S to some extent and approximately 90% of tumors stained for all three cathepsins show high expression levels of at least one of these proteases, which could make this technique less prone to intertumoral heterogeneity.

Although these results are promising, the present study has some limitations. While interpreting these immunohistochemical expression data, one should realize that not only cathepsin expression levels increase during neoplastic transformation and breast cancer progression, but their proteolytic enzymatic activity as well [17]. Because cathepsin-targeted fluorescent imaging agents are activatable molecules, the fluorescence signal these imaging agents produce is dependent on both cathepsin expression level and enzymatic activity. Consequently, a similar cathepsin expression level in two tumors would not necessarily result in a similar degree of imaging agent activation (i.e., fluorescence signal intensity). Accordingly, a tumor with a low cathepsin TIS in this study could prove to have sufficient proteolytic activity to adequately activate an imaging agent in vivo. Another drawback of the current study could be the potential staining of inactive procathepsins, that only become active after auto-activation in the acidic environment of lysosomes [34, 35]. Binding of procathepsins by the primary antibodies used for immunohistochemistry could result in an overestimation of the expression level of the active cathepsin and consequently its imaging agent-activation potential. However, although the exact ratio between procathepsins and cathepsins in breast cancer is unknown, it can be rationalized that a major part of the increased expression levels can be attributed to active cathepsins, as cancer cells upregulate cathepsin expression to facilitate protein turnover and to degrade the extracellular-matrix [14]. Lastly, it should be stated that for accurate detection of (residual) tumor using NIR FI, the target must be upregulated by tumor cells compared to normal tissue, as target expression by healthy tissue could result in false-positive fluorescence signal (i.e. fluorescence in the absence of cancer) [36]. In this study, the cathepsin expression in tumor tissue was not compared to the expression in adjacent healthy breast tissue. However, previous studies have shown that cathepsin expression and activity normal breast tissue is very limited [16, 17, 20].

## Conclusions

The current study investigated the expression of cathepsin B, L, and S in breast cancer TMAs to determine which patient and tumor characteristics are associated with expression of these proteases and which patients might therefore benefit from intraoperative, cathepsin-targeted NIR FI. The expression of at least one of the main cysteine cathepsins, B, L and S in all breast tumor tissues tested—together with the previously reported low cathepsin levels in normal breast tissue – indicates that cathepsin-activatable imaging agents with broad reactivity for these three proteases will likely be effective in the vast majority of breast cancer patients, regardless of molecular subtype and treatment status. Based on our immunohistochemistry results, patients with high grade ER-negative, HER2-positive, or TN tumors might show higher imaging signals.

## Data Availability

The data that support the findings of this study are available from the corresponding author upon reasonable request.
